# Investigating the Attitudes of Adolescents and Young Adults Towards JUUL: Computational Study Using Twitter Data

**DOI:** 10.2196/19975

**Published:** 2020-09-02

**Authors:** Ryzen Benson, Mengke Hu, Annie T Chen, Subhadeep Nag, Shu-Hong Zhu, Mike Conway

**Affiliations:** 1 Department of Biomedical Informatics University of Utah Salt Lake City, UT United States; 2 Department of Biomedical Informatics and Medical Education University of Washington Seattle, WA United States; 3 University Information Technology Infrastructure and Operations University of Utah Salt Lake City, UT United States; 4 Department of Family Medicine & Public Health University of California, San Diego La Jolla, CA United States

**Keywords:** JUUL, electronic cigarettes, smoking cessation, natural language processing, NLP, Twitter, underage tobacco use, tobacco, e-cig, ENDS, electronic nicotine delivery system, machine learning, infodemiology, infoveillance, social media, public health

## Abstract

**Background:**

Increases in electronic nicotine delivery system (ENDS) use among high school students from 2017 to 2019 appear to be associated with the increasing popularity of the ENDS device JUUL.

**Objective:**

We employed a content analysis approach in conjunction with natural language processing methods using Twitter data to understand salient themes regarding JUUL use on Twitter, sentiment towards JUUL, and underage JUUL use.

**Methods:**

Between July 2018 and August 2019, 11,556 unique tweets containing a JUUL-related keyword were collected. We manually annotated 4000 tweets for JUUL-related themes of use and sentiment. We used 3 machine learning algorithms to classify positive and negative JUUL sentiments as well as underage JUUL mentions.

**Results:**

Of the annotated tweets, 78.80% (3152/4000) contained a specific mention of JUUL. Only 1.43% (45/3152) of tweets mentioned using JUUL as a method of smoking cessation, and only 6.85% (216/3152) of tweets mentioned the potential health effects of JUUL use. Of the machine learning methods used, the random forest classifier was the best performing algorithm among all 3 classification tasks (ie, positive sentiment, negative sentiment, and underage JUUL mentions).

**Conclusions:**

Our findings suggest that a vast majority of Twitter users are not using JUUL to aid in smoking cessation nor do they mention the potential health benefits or detriments of JUUL use. Using machine learning algorithms to identify tweets containing underage JUUL mentions can support the timely surveillance of JUUL habits and opinions, further assisting youth-targeted public health intervention strategies.

## Introduction

### Background

Although the overall use of any tobacco product among high school students decreased from 24.2% in 2011 to 19.6% in 2017 [1], overall use increased to 27.1% in 2018 [2] and further to 31.2% in 2019. This increase was primarily influenced by the use of electronic nicotine delivery systems (ENDS). Current use of ENDS among high school students increased from approximately 1.5% in 2011 [1] to approximately 27.5% in 2019 [3]. This rise in ENDS usage appears to be associated with the increasing popularity of the brand JUUL, a compact pod mod device with a disposable or refillable pod typically containing artificial flavors, nicotine salts, and either vegetable glycerin or propylene glycol and whose sales represented 76% of the ENDS market at the end of 2018 [4].

JUUL's popularity stems from 3 main features of the product: appearance, flavors, and nicotine delivery [5,6]. JUUL's sleek “USB-like” design has assisted in the normalization of public ENDS usage and serves to facilitate inconspicuous use in smoking-prohibited areas such as schools and other public places [7]. JUUL was previously available in a variety of youth-appealing flavors, including but not limited to mango, mint, Crème brûlée, and menthol [8]. As of October 2019, JUUL Labs had removed all flavors except for the classic tobacco, Virginia tobacco, and menthol flavors in an attempt to address concerns regarding the appeal of the product to underage users [9].

Where the nicotine concentrations of combustible tobacco products range from 1.5% to 2.5% by weight [10,11], nicotine concentrations in JUUL pods range from 3% (35 mg/mL) to 5% (59 mg/mL) by weight. Although JUUL pods contain a fraction of the total nicotine that a pack of cigarettes does, JUUL users absorb roughly the same amount of nicotine in a single pod as a pack of cigarettes [12]. This suggests that nicotine is being absorbed more efficiently through JUUL pods than through combustible cigarettes — likely a result of cigarette nicotine being combusted into sidestream smoke and JUUL pods’ nicotinic formulation [13]. JUUL pods contain a protonated form of nicotine known as nicotine salts [14], of which the absorption resembles freebase nicotine seen in cigarettes [15,16] but has a smoother feel when inhaled and does not taste as bitter [13,17].

A recent study on youth awareness of JUUL’s nicotine strength demonstrated that 37.4% of adolescents believed JUUL to contain low or medium nicotine strength and 31.4% were unaware of the nicotine strength [18]. These findings suggest that adolescents are unaware of the relatively high nicotine content in a single JUUL pod. Additional research has documented the emergence of JUUL-compatible pods, some containing nicotine concentrations as high as 6.5% [13]. With approximately 90% of adult daily ever smokers beginning before 18 years of age [19] and a lack of public understanding regarding JUUL’s highly concentrated nicotine levels [20], it has been hypothesized that JUUL poses a risk to younger populations for developing nicotine dependency [21,22]. Consequently, nicotine dependency developed in adolescence may result in addiction and potentially a later transition to traditional combustible cigarettes [23]. With the ENDS market rapidly changing in terms of products and patterns of use (ie, pod mods, box mods, vape pens), there are crucial knowledge gaps in understanding underage ENDS use and its consequences [24].

### Studies of JUUL Use Using Social Media

Free and publicly available data obtained from Twitter can provide insight into public perceptions and knowledge of health behaviors. As reported in 2018 and 2019 Pew Research Center surveys, 32% of teenagers between the ages of 13 and 17 years [25] and 44% of adults between the ages of 18 and 24 years [26] use Twitter. Given this age distribution, the platform serves as a promising source of data for understanding adolescent and young adult JUUL use. Previous studies that have utilized Twitter data on JUUL have identified a number of experiences and insights into the product and its users such as the use of JUUL in prohibited environments (eg, schools) [27], the acquisition of JUUL devices and JUUL pods [28], and the correlation between JUUL mentions on Twitter and JUUL sales [29]. In addition to these studies, there is a growing body of work assessing how JUUL is promoted and used by underage individuals on various social media platforms. Not only does the literature suggest a heavy presence of youth JUUL-related content [30], but younger users are also sharing their opinions and experiences with other users and are talking about the various aspects associated with JUUL use [31-33]. However, a large-scale analysis of JUUL-related tweets that utilizes computational methods has, to the best of our knowledge, not been conducted to understand underage patterns of use and perceptions towards JUUL. Using machine learning algorithms to classify tweets allows for the automatic categorization of tweets and eliminates the time-consuming and resource-consuming burden that comes with the labor-intensive manual annotation process. While the application of machine learning to tweets has shown promise in several public health subdisciplines [34,35], these methods are greatly underutilized in ENDS research.

### Objectives

Our primary objective was to further understand salient themes and topics related to JUUL use on Twitter with particular foci on underage JUUL use and health perceptions. Our secondary objective was to use natural language processing (NLP) methods to develop machine learning–based classifiers capable of automatically identifying and evaluating underage-related JUUL mentions as well as positive and negative sentiments towards JUUL. In doing so, we hoped to provide optimally performing classifiers to be further validated and applied to additional work relating to underage JUUL use and its representation on Twitter.

## Methods

### Data Collection

Using the free Twitter application programming interface (API) [36], we collected a sample of 28,590 tweets from July 2018 to August 2019. To query the Twitter API, appropriate JUUL-related keywords were determined with the aid of a tobacco control researcher (SZ). We used the case-insensitive keywords JUUL, Phix, Sourin, myblu, Aspire Breeze, vaping pod, pod mod, and vape pod*,* as these terms are all common to pod mod ENDS devices. As we were primarily interested in the organic perspective of individuals regarding JUUL use, we removed all retweets from the dataset. After retweet removal, our dataset was comprised of 11,556 unique English language tweets.

### Ethical Considerations

This study was determined to be exempt from review by the University of Utah Institutional Review Board (IRB#00076188). To protect user privacy, we refrained from including usernames in this paper. Further, all quotations used are synthesized from multiple examples.

### Manual Twitter Content Analysis

**Figure 1:**
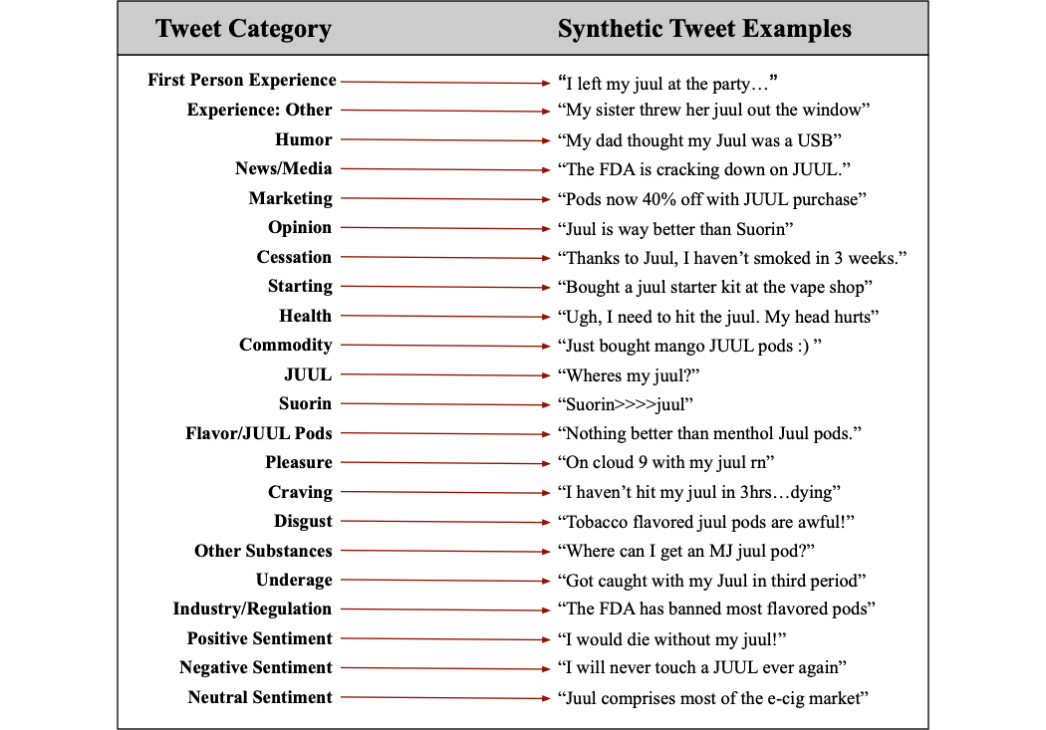
Final categories and synthetic tweet examples, as seen in the manual annotation.

To analyze the various themes of our collected tweets, we carried out a manual annotation process in which we categorized each tweet according to its content. We used the classification scheme developed by Myslin et al [34] for emerging tobacco product Twitter surveillance as a starting point, modifying the classification categories to more appropriately reflect our scope of interest in JUUL. We initially included 39 categories to code for tweet relevancy (ie, whether the tweet was JUUL-related), type, content, and sentiment. At this point, an initial annotation coding round was carried out on 200 tweets to determine the interrater agreement between 2 annotators (RB and MC) and refine the annotation scheme. With consensus among annotators, categories deemed extraneous and irrelevant to our analysis of JUUL (eg, hookah) were excluded from the annotation scheme. Additionally, categories deemed too specific were consolidated with closely related categories. For instance, the separate categories “Industry” and “Policy” were combined to form a singular “Industry and Regulation” category. The final annotation scheme was comprised of 22 categories related to themes of JUUL use, its perceptions among users, and an “Unrelated” category. Our final annotation scheme is available in Multimedia Appendix 1, and synthetic examples of these annotation categories are presented in Figure 1. In an attempt to limit our analysis to JUUL use exclusively, tweets that contained keywords other than JUUL were annotated as “Unrelated” unless the tweet also contained the keyword “JUUL.” Further, we restricted the underage label to those tweets that contained explicit contextual evidence regarding underage elements (eg, “My parents still don’t know I JUUL at school,” “FDA warns of JUUL use in high school,” “For my 16^th^ birthday, I want mango JUUL pods”).

Once the interrater agreement exceeded an acceptable Cohen kappa level [37] (ie, >0.7 [38]), the remaining manual annotation process was carried out by one annotator (RB). Excluding the tweets used for interrater agreement, a total of 4000 tweets were annotated during the manual annotation to ensure there was a sufficient number of tweets for training the machine learning classifiers.

### Data Preprocessing

Using the Natural Language Toolkit (NLTK) [39] – a widely used Python toolkit for analyzing text data – our manually annotated tweets were tokenized using the TweetTokenizer tool. This tool splits characters into individual tokens while also removing punctuation, @ characters, and other extraneous characters. TweetTokenizer is also capable of handling and tokenizing emojis and emoticons. Since these characters are often used in modern text when conveying emotion and sentiment, they are imperative in understanding tweet content. Consequently, we retained emojis and emoticons in the tweets, and they were tokenized as if they were words themselves.

**Figure 2:**
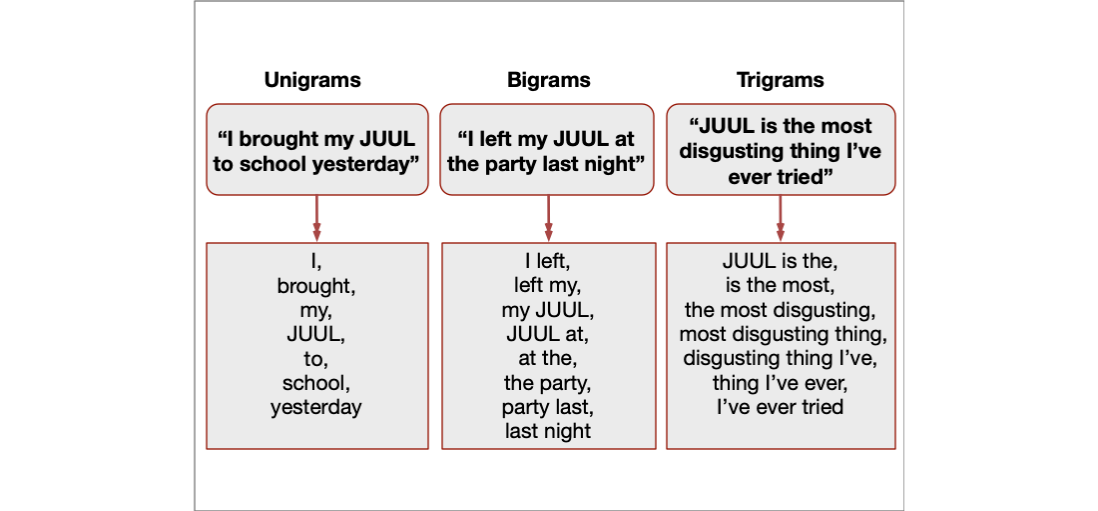
Visualization of n-grams. n-grams can be described as a sequence of n-items, can encode additional semantic content beyond individual words, and once vectorized, can be used as features in machine learning algorithms.

All tokens were then converted into n-gram text sequences. An n-gram (ie, unigram, bigram, trigram) is a contiguous sequence of *n* features used in NLP to transform raw text into features that can be readily processed by a machine learning algorithm (Figure 2).

### Machine Learning Classification

**Figure 3:**
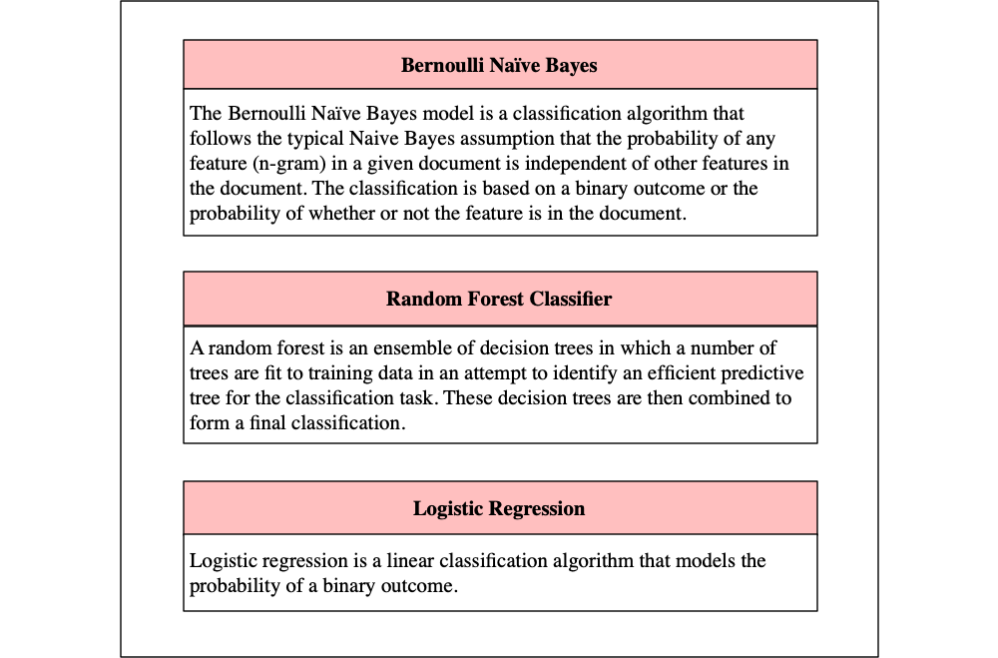
Brief descriptions of the 3 machine learning algorithms used to classify our annotated tweets.

In an attempt to automatically classify JUUL related tweets, we applied supervised machine learning algorithms to identify tweets related to underage JUUL use, positive sentiment, and negative sentiment. The goal of this machine learning–based approach was to identify a predictive function of the data in which unseen data can be accurately classified as containing either underage JUUL use, positive sentiment, or negative sentiment. The efficient and automatic classification of JUUL-related tweets provides a snapshot into the perceptions and use patterns of JUUL and the potential to scale up the analysis beyond what can be realistically performed by manual annotation alone. The algorithms we used for classification were a logistic regression, Bernoulli naïve Bayes, and random forest classifier. Descriptions of the 3 classification algorithms are available in Figure 3.

These models were selected because of their computational simplicity and efficiency in Twitter-based classification tasks [34,40-42]. The input of each classifier consisted of the most salient features determined by feature selection (ie, a process in which the essential terms for model performance are identified automatically, with the rest being discarded).

This feature selection was carried out using Sci-Kit Learn (sklearn) [43], another Python toolkit that is frequently used for text analysis. The tool SelectKBest was used to compare chi-square statistics for each feature and retain the most discerning features of the dataset. In addition to reducing the chance of overfitting the models, feature selection improves model performance due to the removal of features deemed irrelevant. Once a range of suitable features had been selected, the hyperparameters for each algorithm were optimized. This hyperparameter optimization was carried out with sklearn’s GridSearchCV tool, which iterates through specified model parameters and determines the optimally performing model using 10-fold cross-validation. Finally, we applied the optimally performing model to the remaining unannotated tweets.

The following 4 metrics were used to evaluate the performance of the various models: accuracy, precision (positive predictive value), recall (sensitivity), and F1 score (the harmonic mean of precision and recall). These metrics are standard in NLP and reflect a classifier’s ability to classify the task at hand effectively [44,45]. Our goal was to develop classifiers capable of performing well across all 4 metrics, and all 4 metrics were considered when evaluating overall performance.

## Results

### Manual Twitter Content Analysis

Of the 4000 tweets analyzed during the annotation process, 3152 (78.80%) were relevant to JUUL and explicitly mentioned JUUL or JUUL-related accessories such as JUUL pods and chargers. Of the relevant tweets, the most prevalent category was first person usage or experience (1792/3152, 56.85%). The least prevalent categories were using JUUL as a cessation method (45/3152, 1.43%) and using JUUL for the first time (38/3152, 1.21%). Overall sentiment towards JUUL was more positive (1052/3152, 33.38%) than negative (683/3152, 21.67%), and 1416 tweets (1416/3152, 44.92%) demonstrated neutral sentiment. When excluding news, media, and marketing tweets, positive sentiment towards JUUL slightly increased to 33.91% (941/2775) compared to 19.14% (531/2775) for negative sentiment. Lastly, 216 tweets (216/3152, 6.85%) mentioned potential health benefits or detriments of JUUL usage, and 586 tweets (586/3152, 18.59%) mentioned JUUL pods or flavors. See Table 1 for the proportions and frequencies obtained in the manual annotation.

**Table 1:** Category proportions and frequencies from the manual annotation of tweets (n=3152).

Category^a^	Proportion, %	Frequency	

First-person experience	56.85	1792	
Neutral sentiment	44.92	1416	
Positive sentiment	33.38	1052	
Negative sentiment	21.67	683	
Flavor/JUUL pods	18.59	586	
Opinion	15.96	503	
News/media	9.58	302	
Other substances	9.55	301	
Industry/regulation	8.95	292	
Experience: other	7.99	252	
Health effects	6.85	216	
Underage	6.03	190	
Commodity	4.89	154	
Humor	3.20	101	
Suorin	2.54	80	
Marketing	2.38	75	
Pleasure	2.09	66	
Disgust	1.71	54	
Craving	1.46	46	
Cessation	1.43	45	
Starting	1.21	38	

^a^Categories are not mutually exclusive.

### Machine Learning Classification of Underage JUUL Mentions and Sentiment

**Figure 4:**
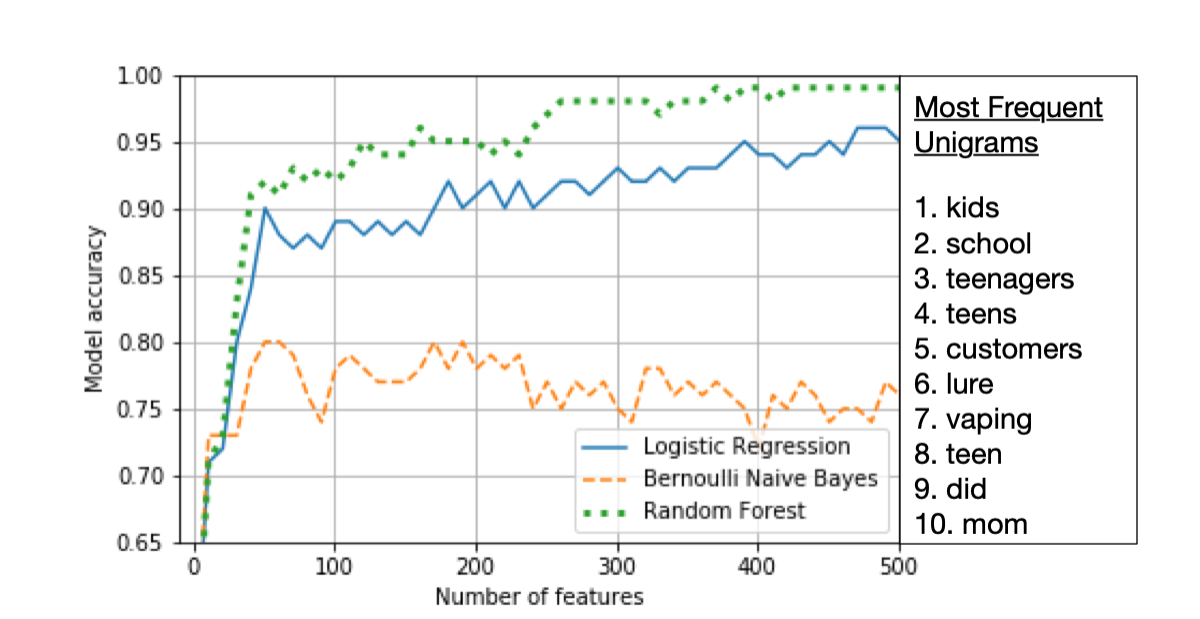
Line plot of model performance at 500 features in classifying underage tweets and the top 10 most discerning features of the underage tweets.

Using supervised machine learning algorithms, we created models to classify underage JUUL mentions and sentiment towards JUUL among Twitter users. To evaluate the different models, we compared the test metrics for all 3 algorithms using the 500 most relevant features for each model (Table 2). In all 3 classification tasks, the random forest model outperformed the logistic regression and Bernoulli naïve Bayes models. When classifying tweets related to underage usage of JUUL, the random forest model yielded a higher accuracy (99% accuracy) when compared to the logistic regression model (94% accuracy) and substantially higher accuracy than the Bernoulli naïve Bayes model (78% accuracy; Figure 4). When comparing the models’ performance for classifying positive and negative tweet sentiment, the random forest model performed considerably better (82% and 91% accuracy, respectively) than the logistic regression model (72% and 78% accuracy, respectively) and the Bernoulli naïve Bayes model (69% and 62% accuracy, respectively). When applying our random forest classifier to additional unseen data (7356 unannotated tweets), our model classified 109 of 7356 tweets as underage-related (1.48%). This proportion is lower than that of the tweets classified as underage-related during the manual annotation process (190/3152, 6.03%), perhaps due to the presence of previously unseen terms related to underage JUUL use.

**Table 2:** Test metrics of the 3 algorithms for all 3 classification tasks as well as average model performance at 500 features for each classification task.

Test metrics and performance	Logistic regression				Bernoulli naïve Bayes				Random forest				
	Acc^a^	*F*	Prec^b^	Rec^c^	Acc	*F*	Prec	Rec		Acc	*F*	Prec	Rec
Underage JUUL use	0.94	0.94	0.95	0.92	0.78	0.71	0.99	0.57		0.99	0.99	0.99	0.99
Positive sentiment	0.72	0.69	0.82	0.69	0.69	0.63	0.83	0.53		0.82	0.82	0.80	0.75
Negative sentiment	0.78	0.77	0.85	0.73	0.72	0.66	0.98	0.50		0.91	0.91	0.90	0.94
Average model performance	0.81	0.80	0.87	0.78	0.73	0.67	0.93	0.53		0.91	0.91	0.90	0.89

^a^Acc: accuracy

^b^Prec: precision

^c^Rec: recall

## Discussion

### Principal Findings

In addition to supporting previous JUUL research using Twitter [27-29], our findings identified critical factors in the understanding and usage of JUUL among Twitter users. In our study, only 1.43% (45/3152) of annotated tweets mentioned using JUUL as a method of smoking cessation. This finding seems incongruent with JUUL’s stated mission of improving the lives of smokers by eliminating combustible cigarette use and replacing it with the — purportedly less harmful — JUUL product [46]. This observation is also inconsistent with the results of a 2019 survey reporting that around 20% of individuals aged 18-24 years initiated JUUL use in an attempt to quit combustible tobacco [47]. Additional research has suggested that youth not only appear to be experimenting with JUUL but are also habitually using the device [48]. Such results, in addition to our findings, suggest that Twitter may be seen as a method of obtaining information to facilitate JUUL use and procurement among youth.

Additionally, only 6.85% (216/3152) of our annotated tweets mention the potential health benefits or detriments of using JUUL, a result consistent with that found by Morean et al [18] and poses the question of whether JUUL users recognize the known effects of high-level nicotine exposure and the potential for developing nicotine dependency and subsequent nicotine addiction. While the long-term effects of JUUL use are yet to be ascertained, there is evidence to support the view that adolescent nicotine exposure may play a significant role in the detrimental alteration of neurochemical, structural, cognitive, and behavioral processes [49].

After removing underage tweets that contained news and media related content, 47% (56/118) of the remaining underage tweets mentioned first-person experiences with JUUL, with 21% (12/56) of those tweets mentioning JUUL pods and flavors — findings consistent with previous literature [28]. Moreover, of those underage first-person mentions, 32% (18/56) contained positive sentiment (eg, “I love my JUUL so much”), compared to 23% (13/56) containing negative sentiment (eg, “Juul is so disgusting”) — a finding that we expected due to the popularity of the pod mod device among youth as compared to other ENDS devices [50].

Although a majority of the tweets that we annotated contained a neutral sentiment towards JUUL (1416/3152, 44.92%), overall tweets contained a more positive sentiment (1052/3152, 33.37%) than negative sentiment (683/3152, 21.67%). And with nearly 20% (586/3152, 18.59%) of the JUUL-related tweets mentioning JUUL pods or flavors, Twitter appears to be regularly used for sharing opinions on various JUUL accessories such as pods or flavors as well as a means to gather information regarding the procurement of such accessories. At face value, it appears that Twitter may be used by individuals to share information about JUUL, thus facilitating its use; additional qualitative research would be necessary to understand the level of exposure of individuals to this content. This finding also suggests the potential for educational campaigns employing Twitter to inform the public about JUUL use, as noted in prior work [16].

Of all the machine learning models we developed, our random forest model performed best in all 3 classification tasks. The performance of the random forest can be primarily attributed to the nature of the algorithm itself. Because a random forest is an ensemble of decision trees containing random subsets of the input features, this algorithm is resilient to outlier data, and the final classification is based on the “majority vote” of the constituent decision trees [51]. Additionally, the random forest’s relatively easy implementation and computational simplicity make it a viable candidate for tobacco control researchers to use in Twitter-based ENDS surveillance.

### Limitations

Our work has some limitations to be considered. First, our data were obtained via the free 1% Twitter API using keyword search rather than the entire Twitter “firehose” dataset; therefore, there is the possibility that not all JUUL-related tweets in the study period were collected. Additionally, our list of keywords (JUUL, Phix, Sourin, myblu, Aspire Breeze, vaping pod, pod mod, and vape pod) is not exhaustive and does not include all pod mod devices available in the United States. We also cannot assume that Twitter users nor their tweets are entirely representative of the general population regarding personal health behaviors.

Second, the frequency of some annotation categories is relatively low, and our models may risk overfitting. In machine learning, overfitting can be described as a model that accurately recognizes patterns and performs well on the training data, but performance decreases when applied to previously unseen data [52]. For instance, our algorithms may fit the data that it was trained on, but if presented with data it has never seen before, it may not be able to maintain this accuracy as the algorithm cannot recognize patterns in the new data.

Additionally, the interpretation of tweet content during the manual annotation process is often subjective due to the brevity of tweet content, lack of grammatical structure, and usage of hyperbole, idioms, and so on. With manual annotation being an inherently interpretive task, we attempted to retain the consistency among our annotations by calculating interrater agreement between annotators, while also focusing on explicit contextual language when assigning labels to tweets.

Finally, the results of this study are preliminary, and in order to derive policy implications from our work, these classification algorithms should be further studied and validated using additional unseen data. Future work should look to apply these classifiers on unlabeled data, conduct error analysis, and refine the algorithms as needed. Pending further validation, these classifiers can be used to automatically categorize large quantities of tweets, allowing researchers to further understand how JUUL is disseminated among youth populations and propose policy change to combat underage ENDS use.

### Conclusions

Our analysis provides a snapshot of the representation of JUUL on Twitter and brings forth several interesting observations for future research endeavors. Our work suggests that the majority of JUUL users on Twitter do not use JUUL as a method of smoking cessation. Additionally, there is a paucity of tweets in which users talk about the potential health effects of using JUUL. Using this manually annotated corpus as training data, we developed 3 supervised machine learning models to accurately classify tweets related to underage JUUL use as well as sentiment towards JUUL. Of the 3 models, our random forest classifier most accurately predicted underage JUUL-related tweets and their sentiment. The application of this algorithm is a novel analytic approach to understanding underage JUUL use on Twitter and, with further research and validation, can promote future research on underage JUUL use patterns as manifested on Twitter.

## Appendix

Multimedia Appendix 1 JUUL-related tweet Annotation Scheme.

## Abbreviations

Acc: accuracy
API: application programming interface
ENDS: electronic nicotine delivery systems
NLP: natural language processing
Prec: precision
Rec: recall
