# Supplementary material for: Investigating the Attitudes of Adolescents and Young Adults Towards JUUL: Computational Study Using Twitter Data
Source: JMIR Public Health Surveill. 2020 Sep 2;6(3):e19975. doi: 10.2196/19975 (PMC7495253; doi:10.2196/19975)
Supplement: Multimedia Appendix 1 [file publichealth_v6i3e19975_app1.pdf]

Appendix 1 — Annotation Scheme

**First person experience**	Spoken/experienced from a personal standpoint.	*My daily routine: Eat, sleep, Juul repeat.*
**Experience: Other**	Spoken/experienced from someone else.	*OMG my mom just bought a juul* 😂
**Unrelated**	Tweets no explicit mention of JUUL	*I lost my vape mod at my buddies house.*
**Opinion**	Personal opinion related to JUUL products, accessories, or JUUL users.	*Juul was the worst thing I have ever tasted.*
**Humor**	Joke or humor related to JUUL.	*Why are there toilets in the JUUL room?*
**News/Media**	News item/story or media related to JUUL.	*Here's my new video on how to blow smoke rings with a juul.*
**Marketing**	Advertising of JUUL product/accessories.	*Buy one pack of JUUL pods get one free at https://....*
**Cessation**	Using JUUL as a tobacco cessation method or the idea of cessation using JUUL.	*On week 2 of quitting smoking thanks to my Juul* ☺
**Starting**	Initiation of JUUL use.	*Just bought my first Juul, let's see what all the hype is!*
**Health**	Health benefits or detriments associated with JUUL use.	*Ever since I bought my JUUL, I get brutal headaches :/*
**Commodity**	The act or details of obtaining or purchasing JUUL devices and/or accessories.	*My brother gave me the rest of his JUUL pods*😭
**JUUL**	Direct reference of the product produced by JUUL Labs Inc.	*Juul has taken over the school!*
**Suorin**	Direct reference of the product produced by Suorin USA.	*I'm thinking of switching from Suorin to JUUL.*
**Flavor/JUUL Pods**	Mention of JUUL flavors, JUUL pods and other JUUL compatible pods.	*Mango Juul pods are their own food group…no debate.*
**Pleasure**	The mention of pleasure resulting from JUUL use.	*This Juul got me feeling spacey!*
**Craving**	Mention of craving JUUL use, oftentimes characterized by symptoms of nicotine withdrawal.	*If I don't juul in the next 5 minutes I might die.*
**Disgust**	Disgust over JUUL use or JUUL users.	*I HATE the smell of JUUL*🤢

**Other Substances**	Mention of other substances such as marijuana, tobacco, alcohol, etc.	*Nothing better than a cold beer and a fresh juul pod*❤️
**Underage**	Mention of JUUL usage by minors or media content portraying this usage.	*Found a juul pod in my 13 year old brothers backpack* 👀
**Industry/Regulation**	Tweets concerning the regulatory, economic and industrial aspects of JUUL.	*The new proposed bill would ban the sale of JUUL to individuals under the age of 21.*
**Positive**	Positive sentiment towards JUUL and its users.	*I can’t live without my JUUL.*
**Negative**	Negative sentiment towards JUUL and its users.	*If he has a JUUL, he’s too young for you sis*
**Neutral**	Neutral sentiment towards JUUL and its users.	*Juul can be purchased in most gas stations.*
